# Comparative Study of Peroneal Tenosynovitis as the Complication of Intraarticular Calcaneal Fracture in Surgically and Non-Surgically Treated Patients

**DOI:** 10.5812/ircmj.11378

**Published:** 2013-10-05

**Authors:** Mahmoud Bahari Kashani, Amir Reza Kachooei, Hengameh Ebrahimi, Mohammad Taghi Peivandi, Sara Amelfarzad, Nastoor Bekhradianpoor, Mehran Azami, Amirreza Fatehi, Golsa Bahari Kashani

**Affiliations:** 1 Mashhad Orthopedic and Trauma Research Center, Faculty of Medicine, Mashhad University of Medical Sciences, Mashhad, IR Iran; 2 Faculty of Medicine, Mashhad University of Medical Sciences, Mashhad, IR Iran; 3 Orthopedic and Trauma Research Center, Mashhad University of Medical Sciences, Mashhad, IR Iran; 4 Department of Orthopedic Surgery, Mashhad University of Medical Sciences, Mashhad, IR Iran; 5 York University, School of Kinesiology & Health Sciences, Toronto, Canada

**Keywords:** Calcaneus, Zygapophyseal Joint, Gissane Angle, Boehelr Angle, Tenosynovitis

## Abstract

**Background:**

Calcaneus has the most fracture prevalence among tarsal bones. About 3/4 of calcaneal fractures are intra-articular fractures with displacement. The majority of calcaneal fractures occur in 21 - 35 year old young men, and that are mostly active people, these fractures cause complete disability for 15 months. Moreover, inappropriate treatment leads to lots of social and economical damages.

**Objectives:**

In this study we compared the incidence and the severity of peroneal tenosynovitis as a complication of non-operative and operative treatment of intra-articular calcaneal fractures. In this study, some other complications of this fracture were also analyzed and the prevalence of the complication was higher in non-operated patients.

**Patients and Methods:**

A total of 140 patients with intra-articular calcaneal fracture were analyzed prospectively. These patients were divided into 2 groups: operated group and non-operated group.

**Results:**

In non-operated group (56 patients), 22 patients were complicated by peroneal tenosynovitis. In operated group (84 patients), 8 patients had the same complication. Statistical analysis revealed that the prevalence, and the severity of this complication in the mentioned groups had a meaningful difference. The results in operated group were much better than the non-operated one.

**Conclusions:**

Although some of the orthopedic surgeons are not interested to manage these fractures surgically and most of them treat these cases conservatively (casting, etc.), in most displaced intra-articular calcaneal fractures, surgical treatment is the method of choice. Moreover, in non-surgical treatment the prevalence of these complications among the patients is more and as a result, inevitable social, occupational and familial damages occur.

## 1. Background

Calcaneal fracture is the most common fracture among tarsal bones, and almost 75% of calcaneal fractures are displaced and intra-articular. Most of the calcaneal fractures occurs in men aged 21 to 45 years old (1), 2% of all fractures include calcaneal fractures, among these 60 - 75% are displaced and intra-articular (2). Peroneal tenosynovitis and tendinitis are commonly seen after non-surgical treatment due to impingement of tendons in the expanded outer wall of the bone.

This may also occur after surgical treatment and it is more common in the previously used, conventional Kocher approach, because in this approach tendons were released from their sheets to provide access to the subtalar joint(3). However, in recent years, as the lateral approach was technically improved, this complication is occasionally seen in the operated patients. Intra-articular calcaneal fractures can be leaded to many complications such as subtalar posttraumatic arthritis, sub-fibular impingement with tenobursitis, subluxation or dislocation of peroneal tendons, reduced calcaneal height, with broader heel, and shoe wear difficulties, sural pain and neuritis of the posterior tibial nerve.

## 2. Objectives

In this study we compared the incidence and the severity of peroneal tenosynovitis as a complication of non-operative and operative treatment of intra-articular calcaneal fractures. In this study, some other complications of this fracture were also analyzed and the prevalence of the complication was higher in non-operated patients.

## 3. Patients and Methods

This prospective study was performed in Imam Reza University Hospital, and Mehr General Hospital in Mashhad, IR Iran, in a two year-period and on the patients hospitalized with intra-articular calcaneal fractures, we compared the operated group and the conservatively treated group for severity and incidence of tenosynovitis of the peroneal tendons as a late complication. To evaluate the frequency of peroneal tenosynovitis in patients with intra-articular calcaneal fractures and compare its prevalence in patients treated non-operatively with surgically treated patients (open reduction and internal fixation) , 140 patients with Saunders type II were evaluated. The patients were randomly divided into 2 groups; A & B. Group A included 56 patients whom intra-articular calcaneal fractures were treated non-operatively. Group B consisted of 84 surgically treated patients.

The method and patients selection was through review of heel radiographs at the time of examination, and CT scanning to determine the type of fracture. Initial proceedings such as posterior splint, limb elevation, pain control, neurovascular examinations, control of compartment syndrome and fracture assessment using heel radiographs and CT-scan were performed. To differentiate the pain of peroneal tenosynovitis from other causes of pain such as calcaneocuboid arthritis or subtalar arthritis; we performed clinical examination, oblique views of the ankle joint in different angles, and MRI to rule out tenoburcitis in peroneal tendons and any cartilage disease which might be occurred at the time of the calcaneus fracture. We also used injection of 1% Lidocaine in to the peroneal tendon sheets.

## 4. Results

56 patients, 48 males and 8 females aged 16 to 45 years old signed up for group A and 84 patients, 62 males and 22 females aged 18 to 50 years old joined to the group B. Some of these patients had bilateral calcaneal fractures, thus the total number of the fractures was more than the number of the patients participating into the study. Among 56 cases in group A, 44 of them had bilateral fracture and 12 had unilateral involvement. In group B, among the 84 cases, 70 of them were involved bilaterally and 14 had unilateral fractures. Therefore, 98 calcaneal fractures were studied. 22 out of the 68 fractures studied in group A were complicated with peroneal tenosynovitis, while this ratio was 8 out of 48 in group B (Table 1). 

**Table 1.** Frequency of Tenosynovitis Among the Study Groups

	Group A		Group B		All Patients		P Value
	Frequency	Percentile	Frequency	Percentile	Frequency	Percentile	
**With tenosynovitis**	22	39.3	8	9.5	30	21.4	
**Without tenosynovitis**	34	60.7	76	90.5	110	78.6	
**Total **	56	100	84	100	140	100	0.000

According to the statistical analysis (P = 0.00), 4 out of those 8 cases who were complicated with peroneal tenosynovitis after surgery, showed subtalar arthritis which was consequently treated with subtalar fusion and reconstruction of lateral calcaneal wall and decompression of peroneal tendon. The frequency of complications associated with tenosynovitis is listed in Table 2. 

**Table 2.** Prevalence of Different Complications Reported in the Study

Complications	Group A		Group B		All Patients	
	Frequency	Percentile	Frequency	Percentile	Frequency	Percentile
**Peroneal tenosynovitis alone**	12	40	2	25	10	45.5
**Peroneal tendon sub luxation**	2	6.7	2	25	0	0
**Peroneal tenosynovitis + subtalar arthritis**	12	40	4	50	8	36.4
**Peroneal tenosynovitis associated with subtalar and talonavicular arthritis**	4	13.3	0	0	4	18.2

Among all 98 patients who underwent surgery for calcaneal fracture, peroneal tenosynovitis was reported in 8 of the cases and their score increased from 65 to 85 according to the AO fas table. Among those 68 patients who underwent nonsurgical modalities, 22 of them were complicated by tenosynovitis and their score increased from 58 to 82.

## 5. Discussion

According to this study, the prevalence of peroneal tenosynovitis after open reduction and internal fixation of calcaneal fracture was significantly different from one after nonsurgical treatments. According to statistical analysis this difference was also considerable between 2 groups. Results of Heckman and colleagues’ study performed in the U.S.(Following up patients after ORIF for 3 & 10 years) support our findings (4). Additionally, results from Raikin and colleagues’ study (Clinical Orthopedics Journal) described ORIF as a less complicative modality for treatment of calcaneal fractures. It is necessary to evaluate ORIF with radiography postoperatively to ensure proper reduction. Despite proper reduction, soft tissue conditions should be noted as a cause of patient dissatisfaction. Therefore, a scoring system was conducted in the study (5). Considering proper evaluations, treatment planning including accurate preoperative assessments, operation at the proper time and anatomic reduction of articular facets and proper fixation of them all will be leaded to significantly less involvement of calcaneal fractures with peroneal tenosynovitis which is virtually an annoying and disabling complication. Because this sort of fracture occurs more commonly in physically active patients, any kind of mismanagement can decrease the patient’s satisfaction and influence their social life(6-9).
